# Prospective study of emergency medicine provider wellness across ten academic and community hospitals during the initial surge of the COVID-19 pandemic

**DOI:** 10.1186/s12873-021-00425-3

**Published:** 2021-03-24

**Authors:** Heather Kelker, Kyle Yoder, Paul Musey, Madison Harris, Olivia Johnson, Elisa Sarmiento, Punit Vyas, Brooke Henderson, Zachary Adams, Julie Welch

**Affiliations:** 1 Department of Emergency Medicine, Indiana University School of Medicine, 1701 N. Senate Blvd, Indianapolis, IN 46202 USA; 2 Indiana University School of Medicine, Indianapolis, IN USA; 3 Department of Psychiatry, Indiana University School of Medicine, Indianapolis, IN USA

**Keywords:** Physician wellness, Well-being, Burnout, Emergency medicine, COVID-19 wellness

## Abstract

**Background:**

While COVID-19 has had far-reaching consequences on society and health care providers, there is a paucity of research exploring frontline emergency medicine (EM) provider wellness over the course of a pandemic. The objective of this study was to assess the well-being, resilience, burnout, and wellness factors and needs of EM physicians and advanced practice providers (e.g., nurse practitioners and physician assistants; APPs) during the initial phase of the COVID-19 pandemic.

**Methods:**

A descriptive, prospective, cohort survey study of EM physicians and APPs was performed across ten emergency departments in a single state, including academic and community settings. Participants were recruited via email to complete four weekly, voluntary, anonymous questionnaires comprised of customized and validated tools for assessing wellness (Well Being Index), burnout (Physician Work Life Study item), and resilience (Brief Resilience Scale) during the initial acceleration phase of COVID-19. Univariate and multivariate analysis with Chi-squared, Fisher’s Exact, and logistic regression was performed.

**Results:**

Of 213 eligible participants, response rates ranged from 31 to 53% over four weeks. Women comprised 54 to 60% of responses. Nonrespondent characteristics were similar to respondents. Concern for personal safety decreased from 85 to 61% (*p* < 0.001). Impact on basic self-care declined from 66 to 32% (*p* < 0.001). Symptoms of stress, anxiety, or fear was initially 83% and reduced to 66% (*p* = 0.009). Reported strain on relationships and feelings of isolation affected > 50% of respondents initially without significant change (*p* = 0.05 and *p* = 0.30 respectively). Women were nearly twice as likely to report feelings of isolation as men (OR 1.95; 95% CI 1.82–5.88). Working part-time carried twice the risk of burnout (OR, 2.45; 95% CI, 1.10–5.47). Baseline resilience was normal to high. Provider well-being improved over the four weeks (30 to 14%; *p* = 0.01), but burnout did not significantly change (30 to 22%; *p* = 0.39).

**Conclusion:**

This survey of frontline EM providers, including physicians and APPs, during the initial surge of COVID-19 found that despite being a resilient group, the majority experienced stress, anxiety, fear, and concerns about personal safety due to COVID-19, putting many at risk for burnout. The sustained impact of the pandemic on EM provider wellness deserves further investigation to guide targeted interventions.

**Supplementary Information:**

The online version contains supplementary material available at 10.1186/s12873-021-00425-3.

## Background

The severe acute respiratory syndrome coronavirus 2 (SARS-CoV-2) aka COVID-19 pandemic has affected nearly every aspect of daily life. Beyond widespread stressors including social isolation, financial hardship, and institutional disruptions [1], health care providers have faced additional workplace demands such as the need to synthesize the deluge of SARS-CoV-2 information, react to rapid clinical practice changes, provide care despite fear of personal illness, uncertainty surrounding access to PPE (personal protective equipment), and staffing changes due to coworkers’ illness or quarantine.

The drastic changes during a pandemic can impact the psychological and physical well-being of frontline healthcare providerss [2, 3]. As seen in prior pandemics, this can lead to negative psychological outcomes including acute and traumatic stress [2, 4–6]. Reports from China and Canada during COVID-19 revealed high rates of distress, insomnia, anxiety, and depression among frontline healthcare providers [3, 4]. These vulnerabilities were further articulated when the United Nations issued a policy brief “COVID-19 and the Need for Action on Mental Health.” [7]

For frontline emergency medicine (EM) providers, including physicians and advance practices providers (e.g., nurse practioners and physician assistants; APPs), wellness is complex, personal, and multifactorial, including constructs like well-being and burnout [8, 9]. Prior to COVID-19, burnout among U.S. EM physicians was already high, in excess of 50% [10–12]. Physician burnout is associated with mental health disorders, interpersonal relationship difficulties, substance abuse, and attrition from the profession [13–20]. Physician burnout negatively impacts quality patient care, patient satisfaction, and the healthcare system as a whole [8, 17–22]. However, physician well-being is more than just the absence of burnout [9, 23]. Well-being involves a myriad of influences on physical and mental health that produce an overall quality of work and life that contributes to the realization of one’s full potential [23].

While studies are still emerging, there is a paucity of research exploring frontline EM provider, including physicians and APPs, burnout and well-being during a global pandemic [3, 4, 24–26] Such information is necessary to inform the decisions of healthcare institutions as they build capacity, support their frontline workforce, and react to the impact of this pandemic. The objectives of this study were to assess the state of EM physician and advanced practice practitioner (APP) well-being, resilience, psychological distress, and burnout during the initial surge of the COVID-19 pandemic, and to identify factors and needs associated with provider wellness in order to guide interventions.

## Methods

### Setting and population

This descriptive, prospective, cohort survey study of EM providers included physicians and APPs (e.g., nurse practitioners and physician assistants) on the frontlines of the COVID-19 pandemic. The eligible study population included 157 employed EM physicians and 56 APPs working in 10 emergency departments within a statewide healthcare system including 7 community hospitals and three academic teaching hospitals in Indiana, United States (U.S.). APPs work collaboratively as a part of the emergency department team and do not have independent practice in Indiana. Participants were recruited via departmental email listservs, with language describing the voluntary and anonymous nature of the surveys, as well as implied informed consent by participation. Nine of the participating hospital dashboards were available and accessed for comparative emergency department patient encounters for 2019 and 2020 during the study dates. The study protocol was reviewed, approved, and deemed exempt by the Indiana University IRB (Protocol l#2003971025) at Indiana University School of Medicine (IUSM).

### Survey development and design

The survey instrument was a combination of customized questions, designed specifically for this study, and three validated tools for assessing wellness (Well-Being Index), burnout (Physician Work Life Study item), and baseline resilience (Brief Resilience Scale) during the initial acceleration phase of COVID-19 (Table 1). After piloting a 22-item draft survey of customized questions to 10 providers from the target population, a modified Delphi technique was used to ensure expert consensus for inclusion of both custom and validated survey tools to assess provider characteristics, COVID-19 related experiences (e.g., quarantine, safety concerns), well-being, burnout, and resilience [38, 39]. Race/ethnicity demographic questions were omitted to ensure respondent anonymity given the lack of racial and ethnic heterogeneity of the population of interest. The final 47-item survey was comprised of multiple choice, scaled rating, and yes-no questions with branching logic to minimize respondent burden (Supplement File 1).

**Table 1:** Survey design

Domain assessed	Tool (validated w/citation, or custom)	Variables/definition	# questions	Collection Timepoint (survey week)			
				3/30–4/6/20	4/7–4/12/20	4/13–4/20/20	4/21–4/27/20
Demographics	Custom	Gender, years out of practice, role etc.	10	X			
COVID Impact	Custom	See Appendix eTable 3	22	X	X	X	X
Wellness	Wellbeing Index [27–31]	Assesses 6 dimensions of distress and well-being from the Mayo Clinic	9	X	X	X	X
Burnout	Physician Work Life Study (PWLS) [32–35]	Single Item Burnout Measure	1	X	X	X	X
Resilience	Brief Resilience Scale [36, 37]	The 6-items of this scale assess resilience, defined as “the ability to bounce back or recover from stress.” It uses a 5-point Likert scale.	6	X			

Well-Being Index (WBI) is a validated, 9-item instrument assessing six dimensions of distress and well-being. Higher WBI total scores [range: − 2 (lowest risk) to 9 (highest risk)] reflect greater distress, lower meaning in work, and lower satisfaction with work–life balance. Scores at or above abnormal risk thresholds (≥3 for physicians and ≥ 4 for APPs) are associated with increased burnout, depression, decreased quality of life, and fatigue. WBI scores correlate other significant events such as medical error and intent to leave the job or profession [27–31].

Physician Work Life Study (PWLS) burnout item is a validated tool asking participants to rate their level of self-defined burnout (1 = “I enjoy my work”; 5 = “I am completely burned out”). Scores were dichotomized (1 or 2 = no burnout; 3 to 5 = burnout symptoms present). Burnout measured via the PWLS predicts high emotional exhaustion, lower work satisfaction, higher self-reported medical error, and greater intent to leave the profession [32–35].

Brief Resilience Scale (BRS) is a validated six-item scale that assesses the ability for the individual to bounce back from stress. Each item is scored on a 1 (strongly disagree) to 5 (strongly agree) Likert scale. Three items are negatively worded and reverse coded. Item scores are averaged for each participant. Average scores correspond to low (1 to 2.99), normal (3 to 4.30), and high (4.31 to 5) resilience [36, 37].

### Survey administration

**Fig. 1:**
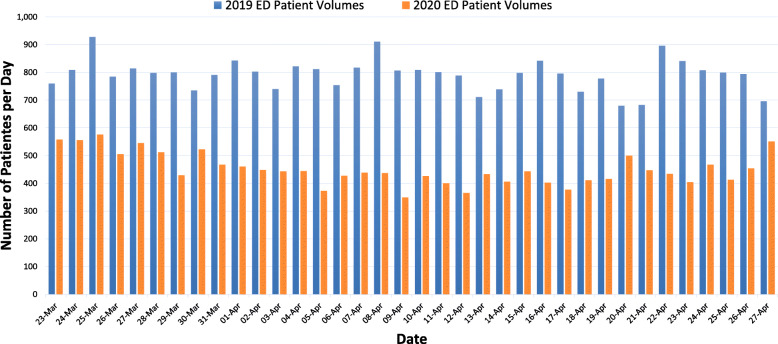
Comparison of 2019 and 2020 Emergency Department Daily Patient Volumes at 9 Study Sites During COVID-19 Surge. During the COVID-19 surge, the daily emergency department patient volumes in 2020 (orange) across nine of the hospital sites studied decreased in comparison to the same dates in 2019 (blue)

Study participation was voluntary and anonymous. Surveys were distributed via departmental listservs and newsletters with permission over four weeks in March and April 2020 (week1, 3/30/20; week 2, 4/7/20; week 3, 4/13/20; week 4, 4/21/20). Participants viewed the study information before starting the survey, and continuation was documentation of consent with no requirement to complete all questions. Each survey was open for 5 days with a 1–2 day wash out period. Nominal incentives (e.g., license plate covers) were offered to randomly selected individuals who self-reported completion of all 4 weeks. Only the study team had direct access to the data. Weekly data were analyzed, with removal of any potentially identifiable open-ended responses, and compiled results were reported to departmental and institutional leadership who designed and implemented interventions to address identified provider needs. Supplement Table 1 and Fig. 1 provides a timeline of selected events and wellness initiatives for EM providers during the study period. The Indiana State Health Department dashboard was accessed for the daily and cumulative positive COVID-19 cases during the study period [40].

### Statistical analysis

Study data were collected and managed using Qualtrics (Qualtrics, Provo, UT) and analyzed using Microsoft Excel for Mac, Version 15.14, and IBM SPSS Statistics for Mac, Version 22.0. Study data are available in the supplementary files or upon request.

Both univariate and multivariate analysis was completed. Frequencies and percentages were summarized by group for categorical variables and continuous variables were summarized by group using the median and range. The proportions of subjects were compared using Chi-square, Fisher’s Exact and Wilcoxon tests. A 5% significance level was used for all tests, *p* < 0.05. Correlation analysis using logistic regression was performed across time points and at the first and last time points to evaluate the associations between PWLS (burnout), the WBI risk, and the odds of responding “Yes” to specific COVID-19 survey questions, reported as odds ratio (OR) with 95% confidence intervals (CI).

## Results

### Demographics

Of the 213 EM providers invited to participate in the study, 157 (74%) were physicians and 56 (26%) were APPs. There were 348 total responses across 4 weeks of data collection. Weekly response rates ranged from 113 (week 1, 53%) to 66 (week 4, 31%). Females (range 54–60%) responded more frequently than males (Table 2). The participant group has similar occupational characteristics as the non-respondent group (Supplement Table 2).

**Table 2:** Demographic and occupational characteristics of survey respondents

Characteristic	No. (%)			
	Week 1 3/30/20–4/6/20	Week 2 4/7/20–4/12/20	Week 3 4/13/20–4/20/20	Week 4 4/21/20–4/27/20
**Total Respondents**	113 (100)	93 (100)	76 (100)	66 (100)
**Work Role**				
Physician	84 (74)	65 (70)	58 (76)	50 (76)
APP	29 (26)	28 (30)	18 (24)	16 (24)
**Gender**				
Male	45 (40)	38 (41)	29 (38)	24 (36)
Female	63 (56)	50 (54)	45 (59)	39 (59)
Prefer not to say	4 (4)	4 (4)	2 (3)	2 (3)
**Age in years**				
20–29	8 (7)	11 (12)	7 (9)	5 (8)
30–39	48 (42)	39 (42)	31 (41)	26 (39)
40–49	26 (23)	18 (19)	18 (24)	16 (24)
50–59	18 (16)	18 (19)	12 (16)	11 (17)
60+	4 (4)	3 (3)	2 (3)	3 (5)
Mean age (SD)	40.8 (SD 9.39)	40.4 (SD 9.61)	40.5 (SD 9.20)	41.4 (SD 9.85)
**Hospital type**				
Academic	74 (65)	54 (58)	44 (58)	39 (59)
Community	31 (27)	33 (35)	28 (37)	23 (35)
Multiple sites	8 (7)	6 (6)	4 (5)	4 (6)
**Work FTE**^**a**^				
< =0.5	10 (9)	6 (6)	6 (76)	6 (9)
0.6–0.7	11 (10)	7 (8)	8 (11)	5 (8)
0.8–0.9	8 (7)	5 (5)	7 (9)	4 (6)
1.0	83 (73)	74 (80)	54 71)	50 (76)
**Years in practice**				
1–5	47 (42)	41 (44)	31 (41)	28 (42)
6–10	21 (19)	15 (16)	13 (17)	7 (11)
11–15	12 (11)	10 (11)	10 (13)	8 (12)
> 16	33 (29)	27 (29)	21 (28)	23 (35)

^a^*FTE* full time equivalent in %, *SD* standard deviation

### Patient volumes and COVID-19 case counts

**Fig. 2:**
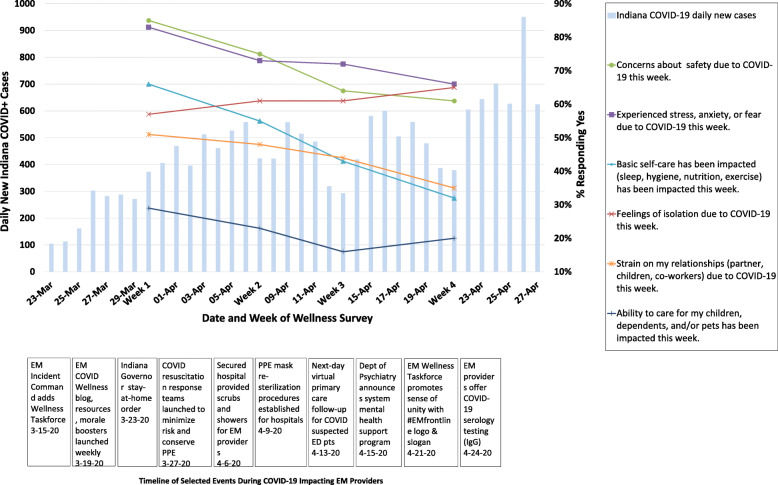
Key Emergency Medicine Provider Wellness Survey Responses and Selected Events as COVID-19 Cases Surge in Indiana. The EM providers (physicians and APPs) experienced greater degrees of distress in all six of the wellness measures included in the figure. The level of distress trended downward during the study period in all areas except feelings of isolation. The daily COVID-19 positive case counts in the Indiana are indicated by the light blue bar graph and reflect the acceleration phase of the pandemic in the state. To offer further context, a timeline of selected events and wellness initiatives are displayed beneath the figure

There was a decrease in the number of overall emergency department patient encounters (i.e., volumes) during the study period in 2020 across nine study sites as compared to 2019 (Fig. 1). In order to provide context for the wellness survey response trends, the daily COVID-19 positive case counts in the state as well as selected events and wellness initiatives during the study period are displayed in Fig. 2.

### Concerns for safety and self-care

Initially, 85% (96/113) of respondents reported acute concerns about their personal or family members’ safety, which decreased to 61% (40/65; *p* = 0.0004) by week four. When polled for ‘interventions that would make you feel safer,’ additional Personal Protective Equipment (PPE) was the most frequent response, followed by hospital provided scrubs and showers (Supplement Table 3). Basic self-care was impacted in 66% (75/113) of EM providers at week one, with significant weekly decreased over the study period (*p* < 0.001). Reduced ability to care for dependents ranged from 29 to 20%, without significant change over time (*p* = 0.21) (Table 3).

**Table 3:** Emergency medicine provider wellness survey responses - COVID specific questions

Question: “yes” responses No. (%)	Week 1^a^ No. = 113	Week 2 No. = 88^b^	Week 3 No. = 75^b^	Week 4 No. = 65^b^	***p***-value^±^ all wks	***p***-value^±^ wk 1 v. 4
Provider Type: Physician	84 (74)	64 (73)	57 (76)	49 (75)	–	–
Provider Type: APP	29 (26)	24 (27)	18 (24)	16 (25)		
**I am concerned about my personal safety and/or the safety of family and dependents due to COVID-19 this week.**	**96 (85)**	**66 (75)**	**48 (64)**	**40 (61)**	0.001	< 0.001
Physician	73 (87)	46 (72)	37 (65)	31 (63)		
APP	23 (79)	20 (83)	11 (61)	9 (56)		
**My basic self-care (sleep, hygiene, nutrition, exercise) has been impacted this week.**	**75 (66)**	**48 (55)**	**32 (43)**	**21 (32)**	< 0.001	< 0.001
Physician	58 (69)	37 (58)	25 (44)	13 (27)		
APP	17 (59)	11 (46)	7 (39)	8 (50)		
**The ability to care for my children, dependents, and/or pets has been impacted this week.**	**32 (29)**	**20 (23)**	**12 (16)**	**13 (20)**	0.22	0.21
Physician	24 (29)	18 (28)	11 (19)	12 (25)		
APP	8 (29)	2 (8)	1 (6)	1 (6)		
**I have experienced stress, anxiety, or fear due to COVID19 this week.**	**94 (83)**	**64 (73)**	**54 (72)**	**43 (66)**	0.06	0.009
Physician	70 (83)	46 (72)	43 (75)	33 (67)		
APP	24 (83)	18 (75)	11 (61)	10 (63)		
**I have experienced strain on my relationships due to COVID-19 this week.**	**57 (51)**	**42 (48)**	**33 (44)**	**23 (35)**	0.26	0.05
Physician	44 (52)	31 (48)	27 (48)	19 (39)		
APP	13 (45)	11 (46)	6 (33)	4 (25)		
**I have experienced personal illness or illness of a loved one this week.**	**23 (20)**	**15 (17)**	**8 (11)**	**7 (11)**	0.20	0.10
Physician	15 (18)	12 (19)	6 (11)	4 (8)		
APP	8 (28)	3 (13)	2 (11)	3 (19)		
**I have experienced additional work responsibilities or hours due to COVID-19 this week.**	**67 (59)**	**43 (49)**	**29 (39)**	**22 (34)**	0.004	0.001
Physician	52 (62)	36 (56)	23 (40)	18 (37)		
APP	15 (52)	7 (29)	6 (33)	4 (25)		
**I have experienced loss of academic/scholarly productivity due to COVID-19 this week.**	**43 (38)**	**27 (31)**	**17 (23)**	**18 (28)**	0.14	0.16
Physician	40 (48)	27 (42)	17 (30)	16 (33)		
APP	3 (10)	–	–	2 (13)		
**I have experienced feelings of isolation due to COVID-19 this week.**	**64 (57)**	**54 (61)**	**46 (61)**	**42 (65)**	0.75	0.30
Physician	49 (58)	39 (61)	36 (63)	32 (65)		
APP	15 (52)	15 (63)	10 (56)	10 (63)		
**I feel supported by my leadership.**	**100 (89)**	**82 (93)**	**74 (99)**	**64 (98)**	0.01	0.02
Physician	72 (86)	58 (91)	56 (98)	48 (98)		
APP	28 (97)	24 (100)	18 (100)	16 (100)		

^a^Week 1 (3/30–4/6/20); Week 2 (4/7–4/12/20); Week 3 (4/13–4/20/20); Week 4 (4/21–4/27/20)

^b^Total No. of respondents completing survey questions. Frequency of missing responses was 5 in week 2, 1 in week 2, and 1 in week 4

^±^*P*-values determined by either Chi-squared or Fisher’s Exact where appropriate

### Provider stress, anxiety, strain, and isolation

Most respondents reported experiencing stress, anxiety or fear at each timepoint, with overall rates decreasing from week one (83%) to week four (66%) (*p* = 0.009). Feelings of isolation were consistently reported by over half of respondents, with no significant change (*p* = 0.30). Approximately half (57/113; 51%) of EM providers experienced strain on their relationships with family, friends or colleagues during the first week of the survey, which dropped to 35% (23/65) by week four (*p* = 0.05) (Table 3).

The self-reported need for mental health resources was low (range: 4–12%). In contrast, 30% (28/113) of respondents reported a desire for stress reduction resources initially, although this level dropped significantly by week four to 9% (4/65; *p* = 0.009). Most respondents reported having access to a mentor, colleague, friend, or family member to help them decompress (range: 81–93%). (Supplement Table 3).

### Work responsibilities and academic productivity

Initially, 59% (67/113) of respondents experienced additional work responsibilities or hours due to COVID-19. This decreased by week four to 34% (22/65; *p* = 0.001). Around one third of respondents experienced loss of academic productivity with no significant change over time (*p* = 0.14). The majority of EM providers felt supported by leadership, which was sustained during the study period, starting at 89% (100/113) and rising to 99% (64/65) by week three (*p* = 0.01) (Table 3).

### Resilience, well-being and burnout scales

The majority of respondents reported normal to high baseline resiliency on the BRS. At week 1, 30% (34/113) of respondents screened positive for burnout on the PWLS and “at risk” on the WBI. While burnout did not significantly change across 4 weeks (*p* = 0.39), the percentage of respondents “at risk” on the WBI decreased significantly to 14% (9/65; *p* = 0.01). (Table 4).

**Table 4:** Emergency Medicine Provider Wellness Survey Responses - Well-Being, Burnout, and Resilience

Question: “yes” responses No. (%)	Week 1^a^ No. = 113	Week 2 ^a^ No. = 88^b^	Week 3 ^a^ No. = 75^b^	Week 4 ^a^ No. = 65^b^	***p***-value^±^ across all weeks	***p***-value^±^ week 1 vs. week 4
Provider Type: Physician	84 (74)	64 (73)	57 (76)	49 (75)	-	-
Provider Type: APP	29 (26)	24 (27)	18 (24)	16 (25)		
**Well-Being Index (at risk)**	**34 (30%)**	**16 (18%)**	**10 (13%)**	**9 (14%)**	0.01	0.02
• Physician	28 (33%)	14 (22%)	10 (13%)	9 (18%)		
• APP	6 (21%)	2 (8%)	2 (11%)	-		
**Physician Work Life Study (burnout measure)**	**34 (30%)**	**20 (23%)**	**15 (20%)**	**14 (22%)**	0.39	0.22
• Physician	25 (30%)	12 (19%)	10 (18%)	9 (18%)		
• APP	9 (31%)	9 (31%)	9 (31%)	9 (31%)		
**Brief Resilience Scale (baseline)**					n/a	n/a
• Physician (% out of No. = 84)						
○ Low resiliency	7 (8%)	-	-	-		
○ Normal resiliency	57 (68%)	-	-	-		
○ High resiliency	20 (24%)	-	-	-		
• APP (% out of No. = 29)						
○ Low resiliency	4 (14%)	-	-	-		
○ Normal resiliency	20 (69%)	-	-	-		
○ High resiliency	5 (17%)	-	-	-		

^a^Week 1 (3/30-4/6/20); Week 2 (4/7-4/12/20); Week 3 (4/13-4/20/20); Week 4 (4/21-4/27/20)

^b^Total No. of respondents completing survey questions. Frequency of missing responses was 5 in week 2, 1 in week 3, and 1 in week 4

^±^*P*-values determined by either Chi-squared or Fisher’s Exact where appropriate

### Associations among variables

In the study, female respondents were almost twice as likely to report feelings of isolation than their male colleagues [odds ratio (OR), 1.95; 95% confidence interval (CI), 1.82–5.88]. Academic hospital-based respondents were twice as likely to report increased work responsibilities compared to providers at community sites (OR, 2.04; 95% CI, 1.27–3.27). Mid-career EM providers were at three times greater odds of having their self-care impacted by the pandemic (OR, 3.03; 95% CI, 1.40–6.58) and over four times more likely to report stress, anxiety, or fear than their early-career counterparts (OR, 4.38; 95% CI 1.27–15.05). Respondents in practice 11–15 years had over twice the odds of screening “at risk” on the WBI than those in practice 1–5 years (OR, 2.50; 95% CI, 1.04–6.04). Working part-time carried twice the risk of burnout on the PWLS (OR, 2.45; 95% CI, 1.10–5.47) (Table 5; Table 6).

**Table 5:** Odds Ratio via logistical regression for provider wellness and demographic correlations

OR (95% CI)	Gender Female vs. Male	Worksite Academic vs. Community	Years Working 6-10 yrs. vs. 1-5 yrs	Years Working 11-15 yrs. vs. 1-5 yrs	Years Working > 15 yrs. vs. 1-5 yrs	FTE 0.5 vs 1.0	FTE 0.6–0.7 vs 1.0	FTE 0.8–0.9 vs 1.0
**WBI (screened at risk)**	0.67 (0.39–1.15)	1.10 (0.61–1.97)	2.12 (0.98–4.60)	2.50 (1.04–6.04)*	1.28 (0.64–2.57)	1.00 (0.35–2.80)	0.44 (0.13–1.50)	0.76 (0.25–2.31)
**PWLS (burn out symptoms)**	1.07 (0.64–1.80)	0.74 (0.44–1.34)	0.77 (0.37–1.62)	1.02 (0.45–2.31)	0.79 (0.44–1.44)	2.45 (1.10–5.47)*	0.41 (0.12–1.40)	1.63 (0.67–4.00)
**I am concerned about safety this week.**	1.4 (0.88–2.35)	1.61 (0.96–2.68)	0.67 (0.35–1.29)	1.78 (0.73–4.36)	1.48 (0.82–2.68)	1.80 (0.66–4.92)	1.88 (0.69–5.11)	0.95 (0.38–2.39)
**My basic self-care has been impacted this week.**	1.13 (0.73–1.75)	1.15 (0.73–1.81)	1.77 (0.95–3.31)	3.03 (1.40–6.58)*	1.21 (0.73–2.01)	1.42 (0.65–3.12)	1.74 (0.79–3.84)	1.77 (0.75–4.20)
**I have experienced stress, anxiety or fear this week.**	1.57 (0.95–2.58)	1.54 (0.92–2.58)	0.94 (0.47–1.86)	4.38 (1.27–15.05)*	1.00 (0.57–1.78)	1.63 (0.60–4.46)	4.79 (1.11–20.67)	0.50 (0.21–1.17)
**I have experienced strain on my relationships this week.**	1.25 (0.80–1.94)	1.30 (0.82–2.06)	1.53 (0.82–2.85)	1.53 (0.74–3.13)	0.72 (0.43–1.21)	1.29 (0.59–2.81)	2.10 (0.96–4.63)	1.09 (0.47–2.52)
**I have experienced personal illness or illness of a loved one this week.**	1.13 (0.61–2.11)	1.92 (0.93–3.94)	1.34 (0.58–3.07)	0.93 (0.33–2.67)	1.30 (0.65–2.61)	2.37 (0.97–5.77)	0.95 (0.31–2.88)	0.54 (0.12–2.39)
**I have experienced additional work responsibilities or hours this week.**	0.69 (0.44–1.07)	2.04 (1.27–3.27)*	1.87 (1.00–3.50)	1.86 (0.91–3.84)	1.48 (0.89–2.47)	2.28 (1.00–5.24)	0.34 (0.14–0.83)	0.54 (0.22–1.31)
**I have experienced feelings of isolation this week.**	1.95 (1.24–3.06)*	1.44 (0.91–2.30)	0.68 (0.36–1.27)	1.13 (0.53–2.40)	0.86 (0.51–1.44)	2.12 (0.87–5.17)	2.71 (1.07–6.88)	0.71 (0.31–1.63)

* statistically significant at *p* < 0.05

*OR* Odds Ratio, *CI* Confidence Interval, *WBI* Well-Being Index, *PWLS* Physician Work Life Study (single item burnout measure), *FTE* full time equivalent employment

**Table 6:** Odds Ratio via logistical regression for provider wellness, well-being and burnout correlations

	Unadjusted		Adjusted**	
	PWLS (Burnout vs. No Burnout	WBI (At risk vs. Not at risk)	PWLS (Burnout vs. No Burnout)	WBI (At risk vs. Not at risk)
**I am concerned about safety this week.**	2.64 (1.36–5.14)*	2.20 (1.10–4.40)*	2.48 (1.24–4.95)*	3.34 (1.50–7.41)*
**My basic self-care has been impacted this week.**	2.71 (1.60–4.61)*	2.80 (1.58–4.96)*	2.80 (1.59–4.90)*	2.67 (1.45–4.90)*
**I have experienced stress, anxiety or fear this week.**	3.08 (1.51–6.29)*	14.97 (3.58–62.53)*	3.33 (1.58–7.04)*	20.44 (4.62–90.41)*
**I have experienced strain on my relationships this week.**	2.76 (1.65–4.61)*	2.99 (1.72–5.23)*	2.66 (1.54–4.59)*	3.00 (1.66–5.45)*
**I have experienced personal illness or illness of a loved one this week.**	1.27 (0.66–2.44)	1.35 (0.68–2.69)	1.05 (0.49–2.24)	1.18 (0.54–2.58)
**I have experienced additional work responsibilities this week.**	1.20 (0.73–1.99)	4.17 (2.31–7.52)*	1.34 (0.77–2.33)	4.08 (2.14–7.78)*
**I have experienced feelings of isolation this week.**	3.27 (1.82–5.88)*	1.79 (1.01–3.18)*	3.53 (1.85–6.71)*	2.23 (1.18–4.22)*

* statistically significant at *p* < 0.05

**controlled for gender, years post training, primary worksite, and current FTE

*WBI* Well-Being Index, *PWLS* Physician Work Life Study (single item burnout measure), *FTE* full time equivalent employment

Respondents who screened positive for burnout on the PWLS or were “at-risk” on the WBI had significantly greater risk in nearly all wellness domains surveyed, including safety concerns, self-care, stress, anxiety, fear, relationship strain, increased work-load, and feelings of isolation due to COVID-19. Results remained unchanged when controlling for gender, years post training, primary worksite, and hiring status [i.e., Full Time Equivalent (FTE)]. EM providers at greatest risk for experiencing stress, anxiety, or fear due to COVID-19 were those who screened “at-risk” on the WBI (OR, 14.97; 95% CI, 3.58–62.53), with greater odds when adjusting for demographic confounders (OR, 20.44; 95% CI, 4.62–90.41) (Table 6).

## Discussion

This prospective longitudinal survey of EM physicians and APPs at academic and community emergency departments was conducted during the acceleration phase of the COVID-19 pandemic in Indiana when cumulative positive COVID-19 cases increased statewide by over 4400% [40]. The study had several interesting findings. First, the majority of frontline EM providers (physicians and APPs) experienced high baseline levels of stress, anxiety, fear, concerns for safety, and relationship strain due to COVID-19. Of note, despite coinciding with the acceleration phase of the pandemic, EM providers reported an *improvement* in each of these domains, although concerns persisted. Second, despite being a resilient group, many providers were at risk for burnout. Third, feelings of isolation endured during the study and were higher for women. Fourth, several subgroups, including women, part-time, mid-career, and academic providers had greater odds of COVID-19 impacting their wellness domains. And lastly, our study was able to identify specific needs of our EM providers (e.g., PPE, scrubs, showers, childcare options, mental health resources) that guided the advocacy work and targeted interventions by our department and institution. (Figure 2 and Supplement Table 1).

The high level of concern about personal safety and the safety of family and dependents found in our study is consistent with the findings from prior pandemics and at other geographical locations affected by COVID-19 [26, 41–44]. Our study adds the early longitudinal perspective that safety concerns among our EM providers steadily improved. This reassuring finding and may be due, in part, to the fact that specific wellness needs were addressed early and resources made available quickly via departmental, institutional, and community initiatives. Examples of interventions include the increased availability of PPE, hospital-supplied scrubs and onsite showers, access to sleep space either on site or at local hotels, and community-based laundry services. As Chen et al. reported, of these interventions, adequate PPE and rest were more important to frontline healthcare providers early in the pandemic to reduce stress than access to a psychologist [45].

Consistent with other studies, a majority of EM providers in our study reported feelings of psychological distress including anxiety, stress, and fear due to COVID-19 [3, 4, 26, 43–45]. In addition, about one third to one half reported increased strain on their relationships (with partner, children, and co-workers). Factors at the individual and systems levels may contribute to these findings. Each EM provider is beholden to the pressures of their collective communities during a pandemic such as fluctuations in childcare and school situations, financial stress, and social isolation. Confounding this, EM providers may experience internal role conflict with regard to their work duties as frontline health care providers and their personal responsibilities to care for family or depedents [2, 45]. Along with these pressures, EM providers often worry about the lack of treatment options or ventilator capacity for patients, bear the fear of infecting family or friends, and face thoughts of their own mortality or that of their colleagues and loved ones [25, 41, 45].

The persistent feelings of isolation experienced in our study is consistent with reports from the 2003 SARS outbreak. During SARS, frontline healthcare workers were at greater risk of feeling isolated than the general public, as well as the associated negative mental health consequences [4]. During COVID-19, the degree of social distancing and isolation is unprecedented on this generation, and the toll it will take on mental health is not fully evident.

Our study found that the well-being of certain subgroups of EM providers (i.e., women, part-time, mid-career, and academic providers) may be at greater risk during a pandemic. For example, in our study, women were twice as likely to affirm feelings of isolation. Other studies have shown that female gender is associated with more severe symptoms of depression, anxiety, or distress due to the COVID-19 pandemic [3, 26, 46–49]. For mid-career and academic providers, it is possible that they had additional non-clinical work responsibilities, personal obligations, and/or work-life conflicts that contributed to their higher reported COVID impact on well-being. It is imperative to consider the unique professional and personal situations of these subgroups in order to target support and resources. Additional investigation into how gender, employment status, or stage of career affects or is affected by the complex circumstances facing frontline EM providers is warranted.

Burnout is a syndrome resulting from chronic workplace stress and is characterized by emotional exhaustion, cynicism or depersonalization from one’s job, and reduced efficacy, which suggests little malleability for positive change under conditions of heightened stress, such as a pandemic [50]. Our study confirmed this, as burnout, using the PWLS item, remained steady at about one quarter of EM providers. Interestingly, this burnout rate did not worsen and is less than the national average for the specialty of EM. This could be in part due to the normal to high baseline resilience scores or from other individual or system factors noted below.

It is curious that during our study the burnout rates remained steady using the PWLS, while provider well-being improved using the WBI, which also contains a burnout item. A likely explanation lies in the difference between the PWLS and WBI scales. The PWLS evaluates the self-determined presence of burnout via a single question. The WBI calculates burnout risk more broadly as one of many domains of wellness. Burnout amongst providers assessed by the WBI may not have changed over time, while other domains of wellness showed improvement, resulting in a lower “at risk” frequency.

Our study suggests that using brief validated scales, such as the PWLS and/or WBI, can provide valuable guidance to institutions before and during pandemics. Of particular note, the EM providers who endorsed burnout on the PWLS single item and screened “at-risk” on the WBI carried greater risks of endorsing concerns about personal safety, impact on dependent care, relationship strain, additional work responsibilities, and feelings of isolation due to COVID. Additionally, EM providers who screened at-risk on the WBI had a twenty times higher odds of reporting stress, anxiety, and fear due to COVID-19. These significant correlations support the utility of these tools in identifying early distress among frontline EM providers and guiding system-based interventions and resource allocation.

The significant improvement in provider well-being on the WBI may be related to both individual and system factors. The timely response and culture of the department and institution may account for mitigating factors, such as the presence of social support, leadership support, safety needs being met (e.g. PPE, scrubs, showers), financial security, childcare options, and access to mental health support. Of particular note, 81–93% of providers reported having a friend, mentor, colleague, or family member to help them decompress, which offers the consistent presence of social support that is found to shield against negative life stress [51, 52]. Additionally, the vast majority of respondents reported feeling supported by leadership (89–99%), which was sustained during the study period. The formation of a departmental wellness taskforce prior to the acceleration phase of the pandemic, whose objective was to evaluate wellness and elicit actionable items during the early stages of the pandemic, may have contributed, in part, to the decreased distress, and may be a key strategy to improve provider well-being.

The literature indicates that system, leadership, and community responses in conjunction with effective communication are crucial prior to and during a pandemic, as this can mitigate negative psychological responses [2]. In our provider group, no frontline EM physicians or APPs experienced salary cuts or furloughs, despite decreased ED volumes, unlike numerous other hospital systems across the country. The tremendous outpouring of appreciation and support for frontline EM providers from the community may also have contributed to the improved wellness factors. Gratitude from patients, families and the community may have offset feelings of burnout and increased job satisfaction [42]. Emergency department volumes significantly decreased during the study period, which may have improved wellness factors. It is also possible, though not measured in this study, that providers’ sense of control and perceived knowledge of the virus improved with time, which has been shown to mitigate negative effects of a pandemic on emotional wellness [53].

### Limitations

This study had several limitations. The study design used an online survey instrument, which is susceptible to response biases including self-selection (voluntary response bias), the sample size (nonresponse bias), as well as the survey length and competing surveys (fatigue bias). Although our response rate is similar to other online survey response rates, it remains a potential limitation [54]. Due to anonymous data collection we could not assess individual-level change over time. Another limitation is the lack of race/ethnicity demographic data, which is needed to further analyze the association of race/ethnicity on provider wellness. Due to the time-sensitive need for the survey, the COVID-specific questions were not validated against other measures, and therefore only have face validity. The WBI asks questions regarding symptoms “over the last month,” however, the instrument was used on a weekly basis and therefore may not be sensitive to that degree of change [55].

## Conclusions

This study of frontline EM physicians and APPs during the initial surge of the COVID-19 pandemic in Indiana found significant levels of stress, anxiety, fear, concerns about safety, and relationship strain, all of which improved but endured. Additionally, while providers were a resilient group, feelings of isolation and burnout persisted, but did not significantly worsen. Well-coordinated departmental and institutional efforts that prioritize wellness can address the central need of frontline workers in ways that may mitigate or buffer acute stressors. The long-term effects of the pandemic on frontline EM providers warrants additional study, along with interventions that may mitigate psychological distress and burnout.

## Supplementary Information

**Additional file 1: Supplement File 1.** “COVID Wellness Survey.” The 47-item survey instrument is a combination of customized COVID-19 specific wellness questions and three validated tools for assessing wellness (Well-Being Index), burnout (Physician Work Life Study item), and resilience (Brief Resilience Scale). The survey is comprised of multiple choice, scaled rating, and yes-no questions with branching logic to minimize respondent burden.

**Additional file 2: Supplement Table 1.** “Timeline of Selected Events and Wellness Initiatives for EM Providers During the Study Period.” Weekly wellness survey data were analyzed and reported to departmental and institutional leadership who designed and implemented interventions to address identified provider needs. The table provides a timeline of selected events and wellness initiatives for EM providers across the study sites during the study period.

**Additional file 3: Supplement Table 2.** “Nonrespondent Characteristics.” The non-respondent group has similar occupational characteristics as the participant group.

**Additional file 4: Supplement Table 3.** “Emergency Medicine Provider Wellness Survey Responses – Identified Needs.” The table provides data from the COVID specific branching questions that identify further wellness needs of the respondents.

## Data Availability

The datasets used during the study are available in the Supplement or from corresponding author upon reasonable request.

## Abbreviations

APP: Advanced practice provider (APP)
SARS-CoV-2: Severe acute respiratory syndrome coronavirus 2 (aka COVID-19)
EM: Emergency medicine
WBI: Well-Being Index
PWLS: Physician Work Life Study
BRS: Brief Resilience Scale
OR: Odds ratio
CI: Confidence intervals
PPE: Personal Protective Equipment
FTE: Full Time Equivalent
